# Vascular injuries following road traffic collisions in a high-income developing country: a prospective cohort study

**DOI:** 10.1186/1749-7922-5-13

**Published:** 2010-05-19

**Authors:** Ali Jawas, Fayez Hammad, Hani O Eid, Fikri M Abu-Zidan

**Affiliations:** 1 Trauma Group, Faculty of Medicine and Health Sciences, UAE University, Al-Ain, United Arab Emirates; 2 Emergency Department, Tawam Hospital; Al-Ain, United Arab Emirates; 3 Department of Surgery Al-Ain Hospital, Al-Ain, United Arab Emirates

## Abstract

**Background:**

The mechanism and pattern of vascular injury vary between different populations. The commonest mechanism of vascular injury in civilian practice is road traffic collisions. We aimed to prospectively study the incidence, detailed mechanism and anatomical distribution of hospitalized vascular trauma patients following road traffic collisions in a high-income developing country.

**Methods:**

Data were collected prospectively on road traffic collision injuries in the whole city of Al-Ain, United Arab Emirates, from April 2006 to October 2007 with full details of mechanism of injury and its relation to sustained injuries.

**Results:**

Out of 1008 patients in the registry, 13 patients had vascular injury, a calculated incidence of 1.87 cases/100 000 inhabitants per year. There were eight car occupants, four pedestrians, and one motorcyclist. Upper limb vascular injuries were the most common anatomical site (n = 4) followed by thoracic aorta (n = 3). All thoracic aortic injuries were acceleration injuries (pedestrians hit by a moving vehicle). None of the eight car occupants was wearing a seatbelt and the majority sustained a front impact deceleration injuries. The median injury severity score, hospital stay, and ICU stay were significantly higher in the vascular injury group compared with nonvascular group (P < 0.0001). Three patients died (23%); two due to severe liver trauma and one due to rupture thoracic aorta.

**Conclusions:**

The incidence of hospitalized vascular injury due to road traffic collisions in Al-Ain city is 1.87 cases/100 000 inhabitants. These injuries occurred mainly in the upper part of the body. Seatbelt compliance of car occupants having vascular injuries was very low. Compliance with safety measures needs more enforcement in our community.

## Introduction

Road traffic death rate in United Arab Emirates (UAE) is one of the highest in the world. It has been recently estimated to be 37.1 per 100 000 population [1]. Furthermore, road traffic collisions (RTC) account for more than 75% of unintentional injury deaths in the UAE [2]. The behavior of drivers and compliance with safety measures in the UAE are completely different from those in developed countries [3,4]. In a recent report; only 25% of drivers who were involved in RTC used seatbelts [4]. We have recently shown that severity of head injury was the most significant factor affecting mortality in patients involved with RTC in our community indicating low compliance with use of seatbelts [5]. Hypotension on arrival was another significant factor affecting RTC mortality [5]. Vascular injuries can be life-threatening and their prompt diagnosis is essential for favorite outcome. The incidence, detailed mechanism, and nature of vascular injuries following road traffic collisions including their anatomical distribution are not well studied in the Middle East. We aimed to prospectively study the incidence, detailed mechanism and anatomical distribution of hospitalized vascular trauma patients following road traffic collisions in a high-income developing country.

## Patients and methods

Data from the RTC Injury Registry of Al-Ain City were collected prospectively from April 2006 to October 2007. The registry involved the two main hospitals in the city (Tawam and Al-Ain Hospitals). Al-Ain City, which is the largest city in the Eastern District of Abu-Dhabi and one of the four largest in the country, had a population of 463,000 inhabitants at the time of the study [6]. The Local Ethics Committee of Al-Ain Health District Area has approved data collection for all road traffic collision trauma patients who were admitted to Al-Ain and Tawam Hospitals or who have died in the Emergency Department.

The data collected included the patient's age, gender and other personal details. In addition it included the type of vehicle (s) involved, the exact mechanism of crash, the use of safety measures, vascular injuries, other injuries, the Injury Severity Score (ISS), the procedures required and the final outcome. The ISS was used as a global measure of injury severity. ISS was calculated manually using the Abbreviated Injury Scale handbook [7,8].

A web-based database was used to enter the data. Data were analyzed with the Statistical Package for the Social Sciences (version 15, SPSS Inc.). Univariate analysis to compare patients with vascular injuries and those without them was done using Mann-Whitney U test for continuous or ordinal data and Fisher's exact test for categorical data. Patients who died were excluded when total hospital stay was calculated. Statistical significance was set at 0.05.

## Results

Out of the 1008 patients who were studied, there were 13 patients with vascular injuries (1.29%). The median age was 26 years (range 2-45). There were 12 males and one female. There were 8 car occupants (3 drivers, 3 front seat passengers and two backseat passengers), 4 pedestrians, and 1 motorcyclist. The full details of mechanism of injury and its relationship to anatomical site of vascular injury are shown in Table 1. None of the car occupants who sustained a vascular injury was wearing a seatbelt. Distribution of the anatomical sites of the vascular injuries is shown in Table 2. Upper limb vascular injuries were the most common followed by the thoracic aorta. The calculated incidence of hospitalized vascular injured patients due to road traffic collisions in Al-Ain City was 1.87 cases/100 000 inhabitants per year.

**Table 1:** Detailed description of mechanism of injury, vascular injuries, and associated injuries.

Patients	Status	Details of mechanism of injury	Vascular injury	Associated injuries
1	Driver, No seatbelt	Saloon car hits another saloon car, right front impact	Femoral artery Right renal artery	Left femur, cervical spine, pelvic fracture, right kidney rupture
2	Driver, No seatbelt	4 wheel hits another 4 wheel, front impact and rollover	Avulsion of axillary artery	Avulsion of brachial plexus, fracture scapula
3	Driver, No seatbelt	4 wheel hits another 4 wheel, rear end impact	Thrombosed left renal artery	Pelvic, femur, and lumbar spine fractures, bilateral lung contusion
4	Front seat passenger No seat belt	Saloon car hits a light post, left front impact	Anterior tibial artery	Skull fracture, subdural haematoma, right pneumothorax, liver laceration
5	Front seat passenger No seat belt	Saloon car hits a 4 wheel, front impact	Main hepatic veins	Lacerated spleen, bilateral lung contusion
6	Front seat passenger No seat belt	Saloon car rollover collision	Right gluteal artery	Pelvic and femur fractures, head injury, liver laceration
7	Back seat passenger No seat belt	Saloon car rollover collision	Brachial artery injury	Supra-chondyler fracture of the right humerus
8	Back seat passenger No seat belt	Saloon car hits a heavy truck, rear end impact	Pelvic vessels	Pelvic fracture
9	Pedestrian	Hit by a saloon car	Thoracic aorta dissection	Bilateral haemothorax, bilateral rib fractures, tibia and fibula fractures
10	Pedestrian	Hit by heavy truck	Portal vein Brachial artery	Fracture humerus, liver laceration, bilateral rib fractures
11	Pedestrian	Hit by a truck	Rupture thoracic aorta	Fracture pelvis, fracture tibia, head injury
12	Pedestrian	Hit by a saloon car	Rupture thoracic aorta	Fracture pelvis, fracture clavicle
13	Motorcyclist No helmet	Rollover	Brachial artery	Humeral fracture

**Table 2:** Anatomical site of vascular injuries.

Anatomical Site	Number
Brachial/axillary artery	4
Thoracic aorta	3
Pelvic vessels	2
Renal artery	2
Femoral artery	2
Portal vein	1
Hepatic veins	1
Anterior tibial artery	1

Total	16

In total, three patients sustained traumatic rupture of the thoracic aorta, one underwent open surgical repair and he died while the others had endovascular aortic stent graft. Both had successful outcome and survived. Three patients needed laparotomy to stop bleeding (two for liver injury and the third for a right nephrectomy), one had a thoracotomy, one had a craniectomy and one had angioembolization for pelvic bleeding.

All five patients who underwent surgical repair for peripheral vascular injury had successful revascularization. The main method for repair was interposition venous graft. One patient of these died secondary to severe bleeding from a liver injury (Patient number 10). The sixth patient underwent surgical exploration with ligation of the tibial vessels (patient number 4). All our vascular injured patients had associated fractures except one.

Table 3 shows the highest Abbreviated Injury Scale (AIS) in the body regions where vascular injuries occurred. The highest AIS in those regions in the vascular injury group were contributed to the vascular injuries. The vascular group had significantly higher AIS in the abdomen and lower limbs (Table 3). The vascular injury group had significantly higher median ISS, total hospital stay and percentage of patients who needed ICU admission (Table 4). Three patients died (23%); two due to vascular injuries of the liver (patients number 5 and 10) and one with aortic arch rupture (patient number 11).

**Table 3:** Median score Abbreviated Injury Scale (AIS) by body region in vascular and non vascular groups.

Area	Vascular group	Non vascular group	P value
Chest	3.5 (1-5)	3 (1-5)	0.07
Abdomen	4 (2-5)	1 (1-4)	0.001
Upper limb	3 (1-3)	2 (1-3)	0.2
Lower limb	3	3 (2-4)	< 0.0001

*P *= Mann Whitney U test

**Table 4:** Severity of injury parameters.

Variable	Vascular injured patients (n = 13)	Non-Vascular injured patients (n = 995)	P value
ISS	29 (range 9-50)	5 (range 1-45)	< 0.0001
Median hospital stay (days)	24 (range 1-73)	3 (range 1-127)	< 0.0001
ICU admission No. (%)	9 (69%)	172 (17%)	< 0.0001

*P *= Mann Whitney U test or Fisher's Exact test as appropriate

## Discussion

The incidence of vascular injury has increased worldwide during the last few years with variation in mechanism and pattern in different populations. The commonest mechanism of injury in civilian practice is road traffic collisions while the increase in penetrating vascular trauma is directly related to the surge of interventional vascular procedures [9].

There have been few studies on vascular injuries from our region. The majority of vascular injuries in Saudi Arabia (57%) were caused by blunt trauma and 91% of those were caused by road traffic collisions [10]. Surprisingly, the commonest cause of vascular injury in Kuwait during the period of 1992-2000 was penetrating firearms and stabbing (43%) and only 23% were caused by road traffic collisions. This may reflect the aftermath of the Gulf War on that community [11]. In contrast RTC accounted for about 40% of all vascular traumas in Ireland and Australia [12,13]. A population based study from Scotland reported an incidence of aortic injuries of 0.3%. Blunt trauma caused 73% of these injuries of which RTC was the most common cause [14]. The majority of vascular trauma in USA, South America and military conflict areas in Europe was penetrating trauma reaching up to 90% in some reports [15-17].

The actual incidence of vascular trauma in most European countries is unknown. Finland has an annual incidence of 1.3 per 100,000 inhabitants while Sweden has an incidence of 2.3 per 100,000 inhabitants [9]. Our incidence of major vascular trauma due to road traffic collisions alone is 1.87 cases/100 000 inhabitants per year. The studies from Sweden and Finland included all vascular injury patients admitted to hospitals. About 20% were caused by blunt trauma. In contrast our study was limited only to hospitalized vascular injury in road traffic collisions. Only 34% of trauma in our community is caused by RTC which indicates that vascular trauma in general is even much higher than Finland and Sweden [18].

It may be argued that the number of patients of this study is small. Nevertheless we think that the data was very accurate as it captured prospectively all injuries in all age groups with their detailed mechanism of injury in a specific population over a specific time.

Analyzing the biomechanics of crashes is important. About 90% of injuries can be clinically predicted if the biomechanics of RTC was well understood [19]. This will help reducing missed injuries. It is important to note that the majority of vascular injuries were in the upper part of the body (upper limb and thorax) similar to other studies [9,12,20]. All thoracic aortic injuries in our study occurred in pedestrians hit by moving vehicles. These are acceleration injuries in which the moving aortic arch is accelerated compared to the fixed part. We have recently shown that injury severity of RTC patients was higher for non vehicle occupants especially pedestrians, who also accounted for most deaths [5]. The risk of thoracic aortic injury was significantly higher with side-impact crashes and particularly if the occupants were unbelted [21] because side impact hits the weak side of the vehicle.

None of our car occupants was wearing seatbelts. If an occupant was not restrained and had a front impact collision, he/she will lean forward [22-24] and may try to protect him/herself with his/her upper limbs leading to their fracture and major vascular injuries of the upper limbs as they cannot tolerate the impact of energy

Defining the incidence and mechanism of vascular trauma would help in adopting preventive strategies and directing resources in this part of the world. Trauma centers should be well equipped with an angiographic suite, interventional radiologists, and a vascular team to optimize clinical outcome of these life-threatening situations.

The most affordable, effective and cheapest way to reduce the burden of injury is prevention [25]. Injury prevention is usually highly cost effective saving both medical costs and lives [26]. We should adopt an epidemiological approach if we are serious in preventing these injuries. This includes providing a practical structure for surveillance, analysis, and prevention of injury [27]. Furthermore, enforcement of the law of seatbelt usage, strict penalties for high speed, and a public educational program are highly needed in our community. We hope that our study is a small step in that direction.

*In *summary, the incidence of hospitalized vascular injury due to road traffic collisions in Al-Ain city is 1.87 cases/100 000 inhabitants. These injuries occurred mainly in the upper part of the body. Seatbelt compliance of car occupants having vascular injuries was very low. Compliance with safety measures needs more enforcement in our community.

## Competing interests

The authors declare that they have no competing interests.

## Ethical approval

The Local Ethics Committee of Al-Ain Health District Area, Al-Ain, (UAE RECA/02/44)

## Authors' contributions

AJ helped in the idea and design of the study, analyzed the data, and wrote the manuscript. FH helped in the idea and writing of the manuscript. HE helped in the idea, design of the study, and collected the data. FAZ had the idea, raised funds for the study, designed the study protocol, and trained the research fellow for data collection, assured the quality of data collected, helped draft the first version of the paper, and repeatedly edited it. All authors have read and approved the final manuscript.
